# Atopic dermatitis is associated with active and passive cigarette smoking in adolescents

**DOI:** 10.1371/journal.pone.0187453

**Published:** 2017-11-01

**Authors:** So Young Kim, Songyong Sim, Hyo Geun Choi

**Affiliations:** 1 Department of Otorhinolaryngology-Head & Neck Surgery, CHA Bundang Medical Center, CHA University, Seongnam, Korea; 2 Department of Statistics, Hallym University, Chuncheon, Korea; 3 Department of Otorhinolaryngology-Head & Neck Surgery, Hallym University College of Medicine, Anyang, Korea; Centre for Addiction and Mental Health, CANADA

## Abstract

**Objective:**

The relationship between passive smoking and atopic dermatitis has previously been reported, but few studies have simultaneously evaluated the association of atopic dermatitis with active and passive smoking.

**Methods:**

The relationships between atopic dermatitis and active and passive smoking were evaluated in Korean adolescents. We used a large, representative, population-based survey (The Korea Youth Risk Behavior Web-based Survey) conducted in 2011 and 2012. Active smoking was classified into 3 groups (0 days, 1–19 days, and ≥ 20 days/month). Passive smoking was categorized into 3 groups (0 days, 1–4 days, and ≥ 5 days/week). Atopic dermatitis diagnosed by a medical doctor either during the past 1 month or during the participant’s lifetime was surveyed. Age, sex, obesity status, region of residence, economic level, and parental educational level of the participants were adjusted as confounders. Adjusted odds ratios (AORs) and 95% confidence intervals (CI) were calculated using multiple logistic regression analysis with complex sampling.

**Results:**

A total of 6.8% (10,020/135,682) of the participants reported atopic dermatitis during the last 12 months. Active smoking was significantly associated with atopic dermatitis (previous 12 months) (AOR [95% CI] of smoking ≥ 20 days/month = 1.18 [1.07–1.29]; 1–19 days/month = 1.11 [0.99–1.23], P = 0.002). Passive smoking was also related to atopic dermatitis (previous 12 months) (AOR [95% CI] of smoking ≥ 5 days/week = 1.12 [1.05–1.20]; 1–4 days/week = 1.08 [1.03–1.13], P < 0.001).

**Conclusion:**

Atopic dermatitis was significantly associated with active and passive smoking in Korean adolescents.

## Introduction

Atopic dermatitis is a common occurrence worldwide, with a prevalence of 20% in young Americans and 29% in young Koreans [1,2]. Numerous studies have explored the modifiable factors of atopic dermatitis including smoking. Although the adverse effects of smoking on asthma have been well documented in adolescents, the implications of smoking on atopic dermatitis are controversial. Several studies have demonstrated that atopic dermatitis was highly correlated with passive smoking, especially when infants were exposed to passive smoking during prenatal or early neonatal periods by their mother [2,3]. Children who were exposed to passive smoking during prenatal or neonatal periods showed a higher odds ratio (OR) (2.06) of atopic dermatitis in a cross-sectional study [2]. In another cross-sectional study, atopic dermatitis was estimated to be 1.97 times more likely to occur per 100 ng/mg increase in the urine cotinine level in children exposed to passive smoking [3]. Prospective birth cohort studies demonstrated no significant correlation between prenatal smoking and atopic dermatitis [4,5]. Several cross-sectional studies have shown a lower prevalence of allergic sensitization in smokers [6,7].

These contradictory findings may be the result of disparities in study design and covariables adjusted for in the analysis. For instance, the results of a few prospective cohort studies, which reported negative or non-significant correlations between atopic dermatitis and passive smoking, may have involved a small study population or exhibited selection bias due to the inclusion of restricted residential regions and a lack of compliance to the survey [4,5,8]. Additionally, because atopic dermatitis generally develops in young individuals and it is rare of children to smoke, the majority of studies have focused on the effects of passive smoking on atopic dermatitis in children.

The purpose of this study was to explore the associations of active and passive smoking with atopic dermatitis in a large, representative Korean adolescent population. Few studies have simultaneously considered passive smoking and active smoking. The present study is one of the largest population-based surveys using numerous adjusted variables. We considered numerous potential confounders including age, sex, obesity, region of residence, economic levels, and education levels of the participants’ parents using multiple logistic regression analysis.

## Materials and methods

### Study population and data collection

The Institutional Review Board (IRB) of the Centers for Disease Control and Prevention of Korea (KCDC) approved this study (2014-06EXP-02-P-A). Written informed consent was obtained from each participant prior to the survey. Because this web-based survey was performed at a school with many participants, informed consent from their parents was exempted. This consent procedure was approved by the IRB of the KCDC.

This cross-sectional study used the data from the Korea Youth Risk Behavior Web-based Survey (KYRBWS). This study covers the entire nation using statistical methods based on designed sampling and adjusted weighted values. The surveys evaluated data from South Korean adolescents using a stratified, two-stage (schools and classes) clustered sampling based on data from the Education Ministry. Sampling was weighted by statisticians, who performed post-stratification and considered the non-response rates and the extreme values. The results of the KYRBWS, conducted in 2011 and 2012, were analyzed. In 2011, 800 middle schools and 800 high schools were selected (participation rate = 95.5% [75,643/79,202]). In 2012, 797 middle schools and 800 high schools were selected (participation rate = 96.4% [74,186/76,980]). The detailed sampling methods are described on the KYRBWS website [9]. The data were collected by the KCDC. Korean adolescents from the 7^th^ through 12^th^ grades completed the self-administered questionnaire voluntarily and anonymously. The validity and reliability of the KYRBWS have been documented by other studies [10,11].

**Fig 1:**
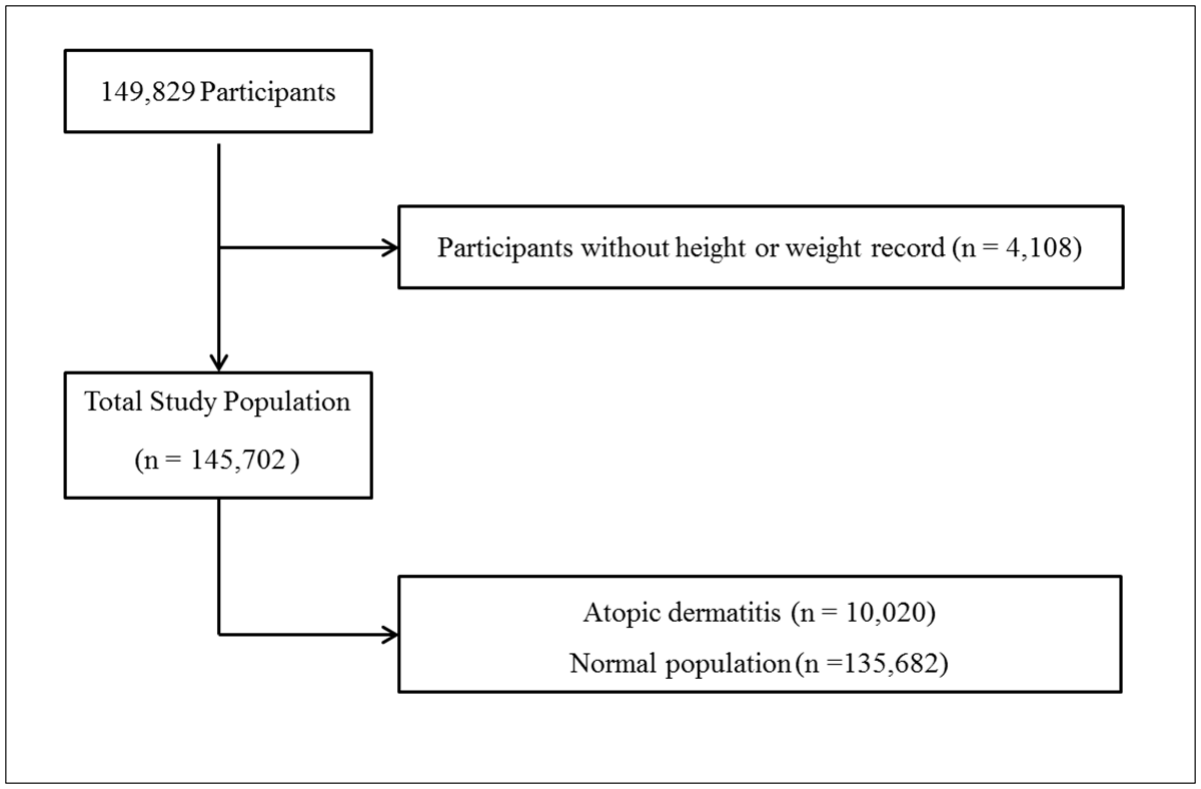
A schematic illustration of participant selection in the present study. Among a total of 149,829 participants, participants without height or weight (n = 4,127) were excluded. The data for the 145,702 participants were analyzed.

Of a total of 149,829 participants, those who did not record their height or weight (4,127 participants) were excluded from this study. Finally, 145,702 participants (73,984 males; 71,718 females), ranging in age from 12 through 18 years, were included in this study (Fig 1).

### Survey

The understanding, reliability and validity of each question were evaluated by the KCDC to determine whether the questions met the criteria of the surveys [12]. The days of physical activity included the number of days of exercise, consisting of more than 60 minutes in which the heart beat or respiration were sufficiently increased, during the past 7 days. Obesity was categorized into 4 groups according to the Centers for Disease Control and Prevention guidelines for the body mass index (BMI, kg/m^2^) for children and teens [13]: obese ≥ 95^th^ percentile; overweight ≥ 85^th^ percentile and < 95^th^ percentile; healthy weight ≥ 5^th^ percentile and < 85^th^ percentile; and underweight < 5^th^ percentile. The region of residence was divided by administrative district into 3 groups: large city; small city; and rural area. The economic level was divided into 5 levels from the highest to the lowest level. The parent education level was divided into 4 groups: graduated college or higher; graduated high school; graduated middle school or less; and unknown or no parent. Participants who did not know the educational levels of their parents or who had no parents were not excluded because this could increase the missing values among participants with relatively low economic levels.

To measure active smoking, the participants were asked the following question: “In the last 30 days, how many days did you smoke at least one cigarette?” Active smoking was divided into 3 groups: 0 days per month; 1–9 days per month; and ≥ 20 days per month. To measure passive smoking, the participants were asked the following question: “In the last 7 days, how many days have you been together with another person (family member or guest) who smoked in your house?” Passive smoking was divided into 3 groups: 0 days per week; 1–4 days per week; and ≥ 5 days per week. To measure E-smoking, the participants were asked the following question: “In the last 30 days, have you smoked an E-cigarette?” E-smoking was divided into two categories: yes or no.

The participants were asked about their history of atopic dermatitis diagnosed by physician as previous studies [14]. The participants were asked the following questions about their history of atopic dermatitis in the past 12 months and during their lifetime: “Have you ever been diagnosed with atopic dermatitis by a doctor?” “In the past 12 months, have you been diagnosed with atopic dermatitis by a doctor?” Participants who had a history of diagnosis by a medical doctor were recorded as positive.

### Statistical analysis

The differences in general characteristics according to the atopic dermatitis history (previous 12 months) were calculated using a linear regression analysis with complex sampling for age and days of physical activity; a chi-squared test with Rao-Scott correction for sex, obesity, region of residence, economic level of the household, education level of the father, education level of the mother, active smoking, passive smoking, and E-smoking was conducted.

Passive smoking rates according to active and E-smoking were compared using a chi-squared test with Rao-Scott correction.

The ORs and adjusted ORs (AORs) of active, passive and E-smoking for an atopic dermatitis history (previous 12 months) were calculated using (i) simple logistic regression analysis with complex sampling (unadjusted); (ii) multiple logistic regression analysis with complex sampling adjusted for age and sex (model 1); (iii) model 1 adjusted for physical exercise, obesity, region of residence, economic level, education level of the father, and education level of the mother (model 2); and (iv) model 2 adjusted for active smoking, passive smoking, and E-smoking (model 3). The ORs and AORs of active, passive and E-smoking for atopic dermatitis history (entire lifetime) were also calculated using the same methods.

Two-tailed analyses were conducted; a value of P < 0.05 was considered to be statistically significant, and 95% confidence intervals (CI) were calculated. The weighted values recommended by the KYRBWS were applied, and all results are presented as weighted values. The results were statistically analyzed using SPSS version 21.0 (IBM, Armonk, NY, USA).

## Results

A total of 6.8% (10,020/135,682) of the participants reported atopic dermatitis in the past 12 months (Table 1). The average age of the atopic dermatitis group was 15.0 years, which was not significantly different from that of normal participants (15.0 years) (P = 0.968). The prevalence of active smoking (P = 0.003) and passive smoking (P < 0.001) were significantly different between the normal and atopic dermatitis groups. However, the prevalence of E-smoking was not significantly different between the groups. Other variables including sex, obesity, economic level, and parental education levels were significantly different between the normal and atopic dermatitis groups (all P < 0.001). Days of physical exercise and region of residence were not significantly different between the normal and atopic dermatitis groups.

**Table 1:** General characteristics of participants.

	Normal Participants	Atopic Dermatitis (recent 12 months)	P-value
Total Number, n (%*)	135,682 (93.2)	10,020 (6.8)	
Age, year (SD)	15.0 (1.7)	15.0 (1.7)	0.968
Physical Exercise, day (SD)	1.7 (1.9)	1.7 (1.9)	0.079
Sex, n (%*)			<0.001†
Male	69,673 (94.2)	4,311 (5.8)	
Female	98,073 (92.0)	8,424 (8.0)	
Obesity, n (%*)			<0.001†
Underweight	9,052 (94.1)	573 (5.9)	
Healthy	108,641 (93.2)	7,955 (6.8)	
Overweight	14,007 (92.2)	1,159 (7.8)	
Obese	3,982 (92.3)	333 (7.7)	
Region, n (%*)			0.258
Large City	61,952 (93.2)	4,533 (6.8)	
Small City	57,238 (93.0)	4,323 (7.0)	
Rural Area	16,492 (93.2)	1,164 (6.8)	
Economic level, n (%*)			<0.001†
Highest	8,459 (93.6)	587 (6.4)	
Middle High	32,117 (93.4)	2,302 (6.6)	
Middle	64,556 (93.3)	4,579 (6.7)	
Middle Low	23,974 (92.5)	1,942 (7.5)	
Lowest	6,576 (91.4)	610 (8.6)	
Education, Father, n (%*)			<0.001†
Unknown	23,320 (94.0)	1,522 (6.0)	
Middle School	6,223 (93.3)	417 (6.7)	
High School	47,171 (92.9)	3,580 (7.1)	
College, or over	58,968 (92.9)	4,501 (7.1)	
Education, Mother, n (%*)			<0.001†
Unknown	22,697 (94.2)	1,421 (5.8)	
Middle School	6,193 (93.1)	437(6.9)	
High School	61,072 (93.0)	4,623 (7.0)	
College, or over	45,720 (92.9)	3,539 (7.1)	
Active Smoking, n (%*)			0.003†
0 day a month	120,374 (93.2)	8,784 (6.8)	
1–19 days a month	6,454 (92.9)	514 (7.1)	
≥ 20 days a month	8,854 (92.6)	722 (7.4)	
Passive Smoking, n (%*)			<0.001†
0 day a week	85,985 (93.4)	6,061 (6.6)	
1–4 days a week	33,541 (92.8)	2,571 (7.2)	
≥ 5 days a week	16,156 (92.2)	1,388 (7.8)	
Electronic Cigarettes, n (%*)			0.685
No	124,301 (93.1)	9,145 (6.9)	
Yes	11,381 (93.1)	875 (6.9)	

* Estimated prevalence adjusted recommended weighted value.

^†^Chi-square test with Rao-Scott correction, Significance at P < 0.05.

Active smoking and E-smoking were significantly related to passive smoking (both P < 0.001) (Table 2). Thus, we adjusted for active smoking, E-smoking, and passive smoking in addition to other variables (model 3). Active smoking was significantly associated with atopic dermatitis (previous 12 months) and exhibited a dose-response relationship when adjusted for age and sex (model 1) and other variables (model 2), although no significant relationship was observed in the unadjusted model (Table 3). These significant relationships between active smoking and atopic dermatitis were maintained even after adjustments for passive smoking and E-smoking (AORs of model 3 [95% CI] for ≥ 20 days per month = 1.18 [1.07–1.29] and for 1–19 days a month = 1.11 [0.99–1.23], P = 0.002). Passive smoking demonstrated consistently significant relationships with atopic dermatitis (previous 12 months) in unadjusted model 1, model 2, and model 3 (AORs of model 3 [95% CI] for ≥ 5 days per week = 1.12 [1.05–1.20] and for 1–4 days a week = 1.08 [1.03–1.13], P < 0.001). E-smoking showed a significant association with atopic dermatitis only in model 1 and model 2 (AORs of model 2 = 1.14, 95% CI = 1.06–1.23, P < 0.001). After adjusting for active and passive smoking (model 3), E-smoking did not show a statistically significant relationship with atopic dermatitis.

**Table 2:** Passive smoking rates according to active and electronic cigarette smoking.

	Passive Smoking			P-value
	0 day a week	1–4 days a week	≥ 5 days a week	
Active Smoking, n (%*)				<0.001†
0 day a month	83,994 (91.1)	3,1558 (87.1)	13,606 (77.1)	
1–19 days a month	3,335 (3.6)	2,447 (6.8)	1,186 (6.7)	
≥ 20 days a month	4,717 (5.3)	2,107 (6.1)	2,752 (16.1)	
Electronic Smoking, n (%*)				<0.001†
No	86,183 (93.4)	32,644 (89.8)	14,619 (82.4)	
Yes	5,863 (6.6)	3,468 (10.2)	2,925 (17.6)	

* Estimated prevalence adjusted recommended weighted value.

^†^ Chi-square test with Rao-Scott correction, Significance at P < 0.05.

**Table 3:** Odd ratios of active, passive and electronic cigarette smoking for atopic dermatitis (recent 12 months) using multiple logistic regression analysis with complex sampling (Reference = no smoking).

Smoking	OR	95% CI	P-value
Active Smoking			
Unadjusted			0.088
0 day a month	1.00		
1–19 days a month	1.05	0.95–1.16	
≥ 20 days a month	1.09	1.00–1.18	
Model 1†			<0.001*
0 day a month	1.00		
1–19 days a month	1.14	1.03–1.26	
≥ 20 days a month	1.22	1.12–1.32	
Model 2‡			<0.001*
0 day a month	1.00		
1–19 days a month	1.13	1.02–1.25	
≥ 20 days a month	1.22	1.13–1.33	
Model 3§			0.002*
0 day a month	1.00		
1–19 days a month	1.11	0.99–1.23	
≥ 20 days a month	1.18	1.07–1.29	
Passive Smoking			
Unadjusted			<0.001*
0 day a week	1.00		
1–4 days a week	1.09	1.04–1.15	
≥ 5 days a week	1.20	1.12–1.27	
Model 1†			<0.001*
0 day a week	1.00		
1–4 days a week	1.09	1.04–1.14	
≥ 5 days a week	1.17	1.10–1.25	
Model 2‡			<0.001*
0 day a week	1.00		
1–4 days a week	1.09	1.03–1.14	
≥ 5 days a week	1.15	1.08–1.23	
Model 3§			<0.001*
0 day a week	1.00		
1–4 days a week	1.08	1.03–1.13	
≥ 5 days a week	1.12	1.05–1.20	
Electronic Cigarettes Smoking			
Unadjusted			
No	1.00		
Yes	1.02	0.95–1.09	0.685
Model 1†			
No	1.00		
Yes	1.14	1.06–1.23	<0.001*
Model 2‡			
No	1.00		
Yes	1.14	1.06–1.23	<0.001*
Model 3§			
No	1.00		
Yes	1.03	0.95–1.12	0.436

* Significance at P < 0.05.

^†^ Adjusted for age and sex.

^‡^ Adjusted for age, physical exercise, sex, obesity, region of residence, economic level, educational level of father, and education level of mother.

^§^ Adjusted for age, physical exercise, sex, obesity, region of residence, economic level, educational level of father, education level of mother, active, passive smoking, and electronic cigarettes smoking.

The effects of active, passive, and E-smoking on atopic dermatitis over the participant’s entire lifetime showed comparable results with those of atopic dermatitis for the past 12 months (S1 Table). Passive smoking demonstrated positive relationships with atopic dermatitis over the participant’s entire lifetime. E-smoking was not significantly related to atopic dermatitis over the participant’s entire lifetime.

## Discussion

The prevalence of atopic dermatitis (previous 12 months) was significantly associated with active and passive smoking.

Consistent with previous studies [2,3], passive smoking was significantly related to atopic dermatitis in the present study. Passive smoking primarily (85%) consists of the hazardous sidestream, which arises from the unfiltered burning of the raw cigarette [15]. The adverse health effects of sidestream smoke are comparable or greater than those of mainstream smoke. After sidestream smoke exposure, allergic responses to inhaled allergens may be rapidly provoked and prolonged through the elevations of IgE, IgG1, and eosinophils [16]. Moreover, macrophage functions are impaired by sidestream smoke [17].

The present study showed higher ORs of active smoking than passive smoking for the prevalence of atopic dermatitis. In accordance with the present results, a recent large-scale, cross-sectional study demonstrated higher ORs for atopic dermatitis for active smoking than for passive smoking [18]. Because an active smoker is expected to encounter higher concentrations and larger amounts of smoke fumes, the adverse effects of smoking may be more remarkable than in passive smokers. Additionally, active smokers were more likely to be exposed to prior passive smoking, which could affect the prevalence of atopic dermatitis. Indeed, a previous study demonstrated a 3.62 times higher prevalence of later-onset atopic dermatitis in previously active smoking-exposed children [19]. Although evidence for the pathophysiologic mechanisms is still lacking, smoking may have several plausible effects on atopic dermatitis. Approximately 4,500 chemical components of cigarettes may modulate the immune system [20]. Smokers have demonstrated low serum concentrations and shortened lifespans of pathogen-specific immunoglobulins [21]. Smoking increases the autoantibody levels [22]. These combined effects of smoking could be predicted to affect allergic sensitization. The inflammatory reaction is another plausible mechanism of the effect of smoking on atopic dermatitis. Strong correlations have been found between smoking and various inflammatory markers, such as the white blood cell count, fibrinogen level, plasma viscosity, and high-sensitivity C-reactive protein level [23]. The skin-barrier function can be damaged by toxic substances produced by smoking, such as nicotine and carbon monoxide, which disturb the blood flow and oxygenation of the skin [24]. These disturbances of the skin and associated subcutaneous structures allow allergens to permeate into the skin, which results in atopic dermatitis [2].

In the present study, E-smoking was also quantified, but could not provide clear information on the relationship between E-smoking use and atopic dermatitis. The insignificant association may have originated from a small proportion of the population of E-smoking, which weakens the statistical power. Many of E-smokers were also the active or passive smokers (S2 Table). Thus, the influence of combined smoking status could not be excluded, although these factors were adjusted in this study. In addition, the binary classification without consideration of the amounts of E-smoking exposure may also have contributed to the lack of relationship between E-smoking and atopic dermatitis. Larger populations who practice E-smoking should be explored to determine the effect of E-smoking on atopic dermatitis.

In the present study, 6.8% of the participants had atopic dermatitis. This calculation is lower than the previously reported value of 20% in a prospective cohort study [2]. In that study, the prevalence of atopic dermatitis could be exaggerated due to selection bias and long-term follow-up loss. The KYRBWS showed a high response rate with a small number of excluded participants due to incomplete data (2.8%). Furthermore, our participants were strategically selected among the entire Korean adolescent population. This representativeness combined with the large survey population increased the validity of our results. However, generalizing the present results should be approached with caution due to likely ethnic, sex, and age effects. Due to the cross-sectional study design, the causality could not be elucidated, and the participants could be affected by the healthy smoker bias. Participants with atopic dermatitis may be more prone to respiratory allergic symptoms, which led them to avoid smoking to manage their respiratory symptoms. Although parental atopy could not be accessed in this study, further analyses adjusted asthma and rhinitis (model 4) also demonstrated significant relation between active or passive smoking and atopic dermatitis (S3 Table). The number of cigarette smoked per day had some variations among participants (S4 Table). The prevalence and the frequency of passive smoking could be underestimated in this study due to the self-reported nature of the study. However, the passive smoking exposure in adolescents has been reported to be well represented by the questionnaire survey and the correlation with urinary cotinine levels [25]. Due to the limitation of survey from large cohort population, detailed questionnaires could not be investigated on AD in this study. Additionally, the present study could not assess objective parameters, such as serum IgE levels or urinary cotinine levels. However, the reliability and validity of the smoking survey of the KYRBWS have been estimated to exhibit 78.0% sensitivity, 97.7% specificity, and a kappa value of 0.80 [9].

## Conclusion

Atopic dermatitis in the past 12 months is significantly associated with active and passive smoking in Korean adolescents. These relationships demonstrated positive associations in accordance with the frequency and dose of active and passive smoking.

## Supporting information

S1 Table Odd ratios of active, passive and electronic cigarette smoking for atopic dermatitis (entire life) using multiple logistic regression analysis with complex sampling (Reference = no smoking). (DOCX)

S2 Table Electronic cigarette smoking rates according to active and passive smoking. (DOCX)

S3 Table Odd ratios of active, passive and electronic cigarette smoking for atopic dermatitis (recent 12 months) using multiple logistic regression analysis with complex sampling (Reference = no smoking). (DOCX)

S4 Table General characteristics of participants. (DOCX)
