# Supplementary material for: Atopic dermatitis is associated with active and passive cigarette smoking in adolescents
Source: PLoS One. 2017 Nov 1;12(11):e0187453. doi: 10.1371/journal.pone.0187453 (PMC5665603; doi:10.1371/journal.pone.0187453)
Supplement: S1 Table — (DOCX) [file pone.0187453.s001.docx]

**S1 Table** Odd ratios of active, passive and electronic cigarette smoking for atopic dermatitis (entire life) using multiple logistic regression analysis with complex sampling (Reference = no smoking)

| Smoking | | | OR | 95% CI | P-value |
| --- | --- | --- | --- | --- | --- |
| Active Smoking | | |  |  |  |
|  | Unadjusted | |  |  | <0.001* |
|  |  | 1-19 days a month | 0.85 | 0.80-0.90 |  |
|  |  | ≥ 20 days a month | 0.88 | 0.84-0.93 |  |
|  | Model 1† | |  |  | 0.017* |
|  |  | 1-19 days a month | 0.92 | 0.87-0.98 |  |
|  |  | ≥ 20 days a month | 1.02 | 0.97-1.07 |  |
|  | Model 2‡ | |  |  | 0.010* |
|  |  | 1-19 days a month | 0.94 | 0.89-1.00 |  |
|  |  | ≥ 20 days a month | 1.05 | 1.00-1.11 |  |
|  | Model 3§ | |  |  | 0.011* |
|  |  | 1-19 days a month | 0.94 | 0.88-0.99 |  |
|  |  | ≥ 20 days a month | 1.05 | 0.99-1.11 |  |
| Passive Smoking | | |  |  |  |
|  | Unadjusted | |  |  | 0.003* |
|  |  | 1-4 days a week | 1.04 | 1.01-1.07 |  |
|  |  | ≥ 5 days a week | 1.05 | 1.01-1.10 |  |
|  | Model 1† | |  |  | 0.066 |
|  |  | 1-4 days a week | 1.03 | 1.00-1.06 |  |
|  |  | ≥ 5 days a week | 1.03 | 0.99-1.07 |  |
|  | Model 2‡ | |  |  | 0.003* |
|  |  | 1-4 days a week | 1.04 | 1.01-1.07 |  |
|  |  | ≥ 5 days a week | 1.05 | 1.01-1.09 |  |
|  | Model 3§ | |  |  | 0.003* |
|  |  | 1-4 days a week | 1.05 | 1.02-1.08 |  |
|  |  | ≥ 5 days a week | 1.05 | 1.01-1.09 |  |
| Electronic Cigarettes Smoking | | |  |  |  |
|  | Unadjusted | | 0.86 | 0.82-0.90 | <0.001* |
|  | Model 1† | | 0.99 | 0.94-1.03 | 0.522 |
|  | Model 2‡ | | 1.00 | 0.96-1.05 | 0.855 |
|  | Model 3§ | | 0.98 | 0.93-1.03 | 0.452 |

* Significance at P < 0.05

† Adjusted for age and sex

‡ Adjusted for age, physical exercise, sex, obesity, region of residence, economic level, educational level of father, and education level of mother

§ Adjusted for age, physical exercise, sex, obesity, region of residence, economic level, educational level of father, education level of mother, active, passive smoking, and electronic cigarettes smoking
