# Supplementary material for: Atopic dermatitis is associated with active and passive cigarette smoking in adolescents
Source: PLoS One. 2017 Nov 1;12(11):e0187453. doi: 10.1371/journal.pone.0187453 (PMC5665603; doi:10.1371/journal.pone.0187453)
Supplement: S2 Table — (DOCX) [file pone.0187453.s002.docx]

**S2 Table** Electronic cigarette smoking rates according to active and passive smoking

|  |  | Electronic Cigarette Smoking | | P-value |
| --- | --- | --- | --- | --- |
|  |  | No | Yes |  |
| Active Smoking, n (%*) | |  |  | <0.001† |
|  | 0 day a month | 124,501 (96.3) | 4,567 (3.7) |  |
|  | 1-19 days a month | 4,992 (70.5) | 1,967 (29.5) |  |
|  | ≥ 20 days a month | 3,953 (39.2) | 5,623 (60.8) |  |
| Passive Smoking, n (%*) | |  |  | <0.001† |
|  | 0 day a week | 86,183 (93.4) | 5,863 (6.6) |  |
|  | 1-4 days a week | 32,644 (89.8) | 3,468 (10.2) |  |
|  | ≥ 5 days a week | 14,619 (82.4) | 2,925 (17.6) |  |

* Estimated prevalence adjusted recommended weighted value

† Chi-square test with Rao-Scott correction, Significance at P < 0.05
