# Supplementary material for: Atopic dermatitis is associated with active and passive cigarette smoking in adolescents
Source: PLoS One. 2017 Nov 1;12(11):e0187453. doi: 10.1371/journal.pone.0187453 (PMC5665603; doi:10.1371/journal.pone.0187453)
Supplement: S3 Table — (DOCX) [file pone.0187453.s003.docx]

**S3 Table** Odd ratios of active, passive and electronic cigarette smoking for atopic dermatitis (recent 12 months) using multiple logistic regression analysis with complex sampling (Reference = no smoking)

| Smoking | | | OR | 95% CI | P-value |
| --- | --- | --- | --- | --- | --- |
| Active Smoking | | |  |  |  |
|  | Model 4† | |  |  | 0.026* |
|  |  | 0 day a month | 1.00 |  |  |
|  |  | 1-19 days a month | 1.08 | 0.97-1.20 |  |
|  |  | ≥ 20 days a month | 1.13 | 1.03-1.24 |  |
| Passive Smoking | | |  |  |  |
|  | Model 4† | |  |  | 0.002* |
|  |  | 0 day a week | 1.00 |  |  |
|  |  | 1-4 days a week | 1.07 | 1.02-1.12 |  |
|  |  | ≥ 5 days a week | 1.10 | 1.03-1.18 |  |
| Electronic Cigarettes Smoking | | |  |  |  |
|  | Model 4† | |  |  |  |
|  |  | No | 1.00 |  |  |
|  |  | Yes | 1.03 | 0.95-1.12 | 0.480* |

* Significance at P < 0.05

† Adjusted for age, physical exercise, sex, obesity, region of residence, economic level, educational level of father, education level of mother, asthma history, allergic rhinitis histories, active, passive smoking, and electronic cigarettes smoking. The history of asthma and allergic rhinitis were surveyed in the past 12 months
