# Supplementary material for: Atopic dermatitis is associated with active and passive cigarette smoking in adolescents
Source: PLoS One. 2017 Nov 1;12(11):e0187453. doi: 10.1371/journal.pone.0187453 (PMC5665603; doi:10.1371/journal.pone.0187453)
Supplement: S4 Table — (DOCX) [file pone.0187453.s004.docx]

**S4 Table** General Characteristics of Participants

|  |  | Normal Participants | Atopic Dermatitis  (recent 12 months) | P-value |
| --- | --- | --- | --- | --- |
| Total Number, n (%*) | | 135,682 (93.2) | 10,020 (6.8) |  |
| Active Smoking, n (%*) | |  |  | <0.001† |
|  | < 1 cigarette a day | 123,052 (90.5) | 9,000 (89.9) |  |
|  | 1 cigarette a day | 1,323 (1.0) | 95 (0.9) |  |
|  | 2-5 cigarette a day | 4,876 (3.6) | 372 (3.5) |  |
|  | 6-9 cigarette a day | 3,473 (2.6) | 265 (2.7) |  |
|  | 10-19 cigarette a day | 2,161 (1.7) | 200 (2.1) |  |
|  | ≥ 20 cigarette a day | 806 (0.6) | 88 (0.9) |  |

* Estimated prevalence adjusted recommended weighted value

†Chi-square test with Rao-Scott correction, Significance at P < 0.05
